# Learning From Limited Data: Towards Best Practice Techniques for Antimicrobial Resistance Prediction From Whole Genome Sequencing Data

**DOI:** 10.3389/fcimb.2021.610348

**Published:** 2021-02-15

**Authors:** Lukas Lüftinger, Peter Májek, Stephan Beisken, Thomas Rattei, Andreas E. Posch

**Affiliations:** ^1^ Ares Genetics GmbH, Vienna, Austria; ^2^ Division of Computational Systems Biology, Department of Microbiology and Ecosystem Science, University of Vienna, Vienna, Austria

**Keywords:** machine learning, genomics, antimicrobial resistance, antibiotics, whole genome sequencing (WGS)

## Abstract

Antimicrobial resistance prediction from whole genome sequencing data (WGS) is an emerging application of machine learning, promising to improve antimicrobial resistance surveillance and outbreak monitoring. Despite significant reductions in sequencing cost, the availability and sampling diversity of WGS data with matched antimicrobial susceptibility testing (AST) profiles required for training of WGS-AST prediction models remains limited. Best practice machine learning techniques are required to ensure trained models generalize to independent data for optimal predictive performance. Limited data restricts the choice of machine learning training and evaluation methods and can result in overestimation of model performance. We demonstrate that the widely used random k-fold cross-validation method is ill-suited for application to small bacterial genomics datasets and offer an alternative cross-validation method based on genomic distance. We benchmarked three machine learning architectures previously applied to the WGS-AST problem on a set of 8,704 genome assemblies from five clinically relevant pathogens across 77 species-compound combinations collated from public databases. We show that individual models can be effectively ensembled to improve model performance. By combining models *via* stacked generalization with cross-validation, a model ensembling technique suitable for small datasets, we improved average sensitivity and specificity of individual models by 1.77% and 3.20%, respectively. Furthermore, stacked models exhibited improved robustness and were thus less prone to outlier performance drops than individual component models. In this study, we highlight best practice techniques for antimicrobial resistance prediction from WGS data and introduce the combination of genome distance aware cross-validation and stacked generalization for robust and accurate WGS-AST.

## Introduction

Antimicrobial resistance (AMR) is a rising global threat to human health. To ensure the continued efficacy of antimicrobial compounds, prudent use of this resource is crucial (O’Neill, 2016). Accurate determination of antimicrobial resistance *via* antimicrobial susceptibility testing (AST) is crucial to ensure optimal patient treatment as well as to inform antibiotic stewardship and outbreak monitoring.

In this context, resistance predictions from WGS data may effectively complement phenotypic AST: The time-to-result (TTR) of WGS-based workflows is effectively governed by the continuously decreasing cost and runtime of genome sequencing, while phenotypic testing is ultimately limited by the pathogen’s growth rate (Bradley et al., 2015; Břinda et al., 2018). Machine learning (ML) algorithms are increasingly applied for prediction of AMR from WGS data (WGS-AST). Recently described WGS-AST techniques use nucleotide k-mer representations of genome assemblies or raw sequencing data, attempting to learn differences in k-mer counts or presence/absence patterns that correlate with shifts in susceptibility to a target antibiotic (Drouin et al., 2016; Aun et al., 2018; Nguyen et al., 2018a; Drouin et al., 2019). This data-driven approach does not require expert knowledge of AMR mechanisms or prior information on AMR genes, and can thus also be applied towards learning of models for novel antibiotics and unknown resistance mechanisms. Other representations of genomic data, such as amino acid k-mers or protein variants have been used for WGS-AST model training as well (Kim et al., 2020; Valizadehaslani et al., 2020).

Challenges arise, however, when learning is not based on features derived from validated, curated AMR markers for the resistance phenotype in question. For example, the significant impact of population structure when applying ML algorithms to WGS-AST data has been noted before (Hicks et al., 2019). Performance of ML models evaluated on isolates from the same experiment as the training data tends to be significantly higher than performance on isolates sampled from independent data sources. Due to limited availability of WGS data coupled with AST information, the performance of WGS-AST models is usually evaluated by cross-validation (CV). Most commonly this is performed using a random splitting criterion, i.e., by dividing samples randomly (Davis et al., 2016; Nguyen et al., 2018a; Drouin et al., 2019). Performance measures obtained by random CV can however only be assumed valid for the larger population if the sample-generating process yields approximately independent and identically distributed (i.i.d.) samples (Ruppert, 2004). This assumption is violated in data points generated by evolutionary processes, which are correlated as a function of the recency of their last common ancestor. This includes, for example, data pertaining to gene function (Tabe-Bordbar et al., 2018) or protein structure (AlQuraishi, 2019), but also whole genomes. By random splitting, similar samples in an existing dependence structure, e.g., evolutionary distance, may be split into the training and test set of CV. This causes the model to overfit by learning features that are spuriously correlated with the phenotype, features which are also present in the test set due to the violated assumption of independence. (Roberts et al., 2017) For example, k-mers mapping to the replication machinery of a resistance cassette-carrying plasmid vector may be highly correlated with resistance due to the prevalence of the plasmid in resistant isolates, despite not contributing to resistance itself. A model overfit to this population by inclusion of such spurious correlations may fail unexpectedly on a population of isolates where the resistance cassette has integrated into the genome. Biological datasets with low sample count but a high number of features further increase the potential of dependence structures and the risk of overfitting (Clarke et al., 2008), and are known to be susceptible to overestimation of model performance by random CV (Roberts et al., 2017).

Ultimately, applying a trained model to multiple large and independently sampled datasets is the gold standard for gauging model generalizability, though this is currently impractical for WGS-AST. To estimate generalization performance in the absence of additional data, blocking CV techniques can be used. Blocking CV seeks to split data into pre-defined similar groups of samples, thus reducing the splitting of dependence structures into the training and test sets of CV (Valavi et al., 2019).

Another significant challenge in achieving robust WGS-AST models with high predictive accuracy is selection of an appropriate learning algorithm. High dimensionality and a low number of training samples constrain the selection of suitable choices. In this study we selected three established learning algorithms which have previously been applied to the WGS-AST problem, and exhaustively benchmarked them across a set of five clinically relevant pathogens (*A. baumannii, E. coli, K. pneumoniae, P. aeruginosa and S. aureus*) and a total of 77 species-compound combinations. We also investigated the possibility of improving model accuracy and robustness by ensembling different learning algorithms such as majority vote and stacked generalization (Wolpert, 1992). This commonly used set of techniques has, to the best of our knowledge, not been explored in the context of antimicrobial resistance prediction from WGS data.

## Results

### Random CV May Overestimate WGS-AST Model Generalizability

To assess the impact of data splitting techniques on performance estimates of WGS-AST models, we trained extreme gradient boosting (Chen and Guestrin, 2016) models under random and genome distance-aware CV. Genome distance-aware CV attempts to improve independence of test sets by segregating samples based on a known dependence structure in the data, namely genome similarity (see Methods). This mirrors the application of the trained model towards independently sampled datasets, in the absence of actual new data.

Genome assemblies coupled with AST information were obtained from public databases (see Methods) for five human pathogens (*A. baumannii*, *E. coli*, *P. aeruginosa*, *K. pneumoniae* and *S. aureus*) and a total set of 77 organism/compound combinations. Data was split into 5 CV folds by either a random or genome distance-aware splitting criterion. Random CV splitting was repeated 10 times while varying the random seed to enable significance estimation (see **Supplementary Methods Section 3**). Extreme gradient boosting (XGB) machine learning models were trained on nucleotide k-mer representations of each of the resulting training sets (see Methods) and evaluated on the corresponding test sets.

**Figure 1:**
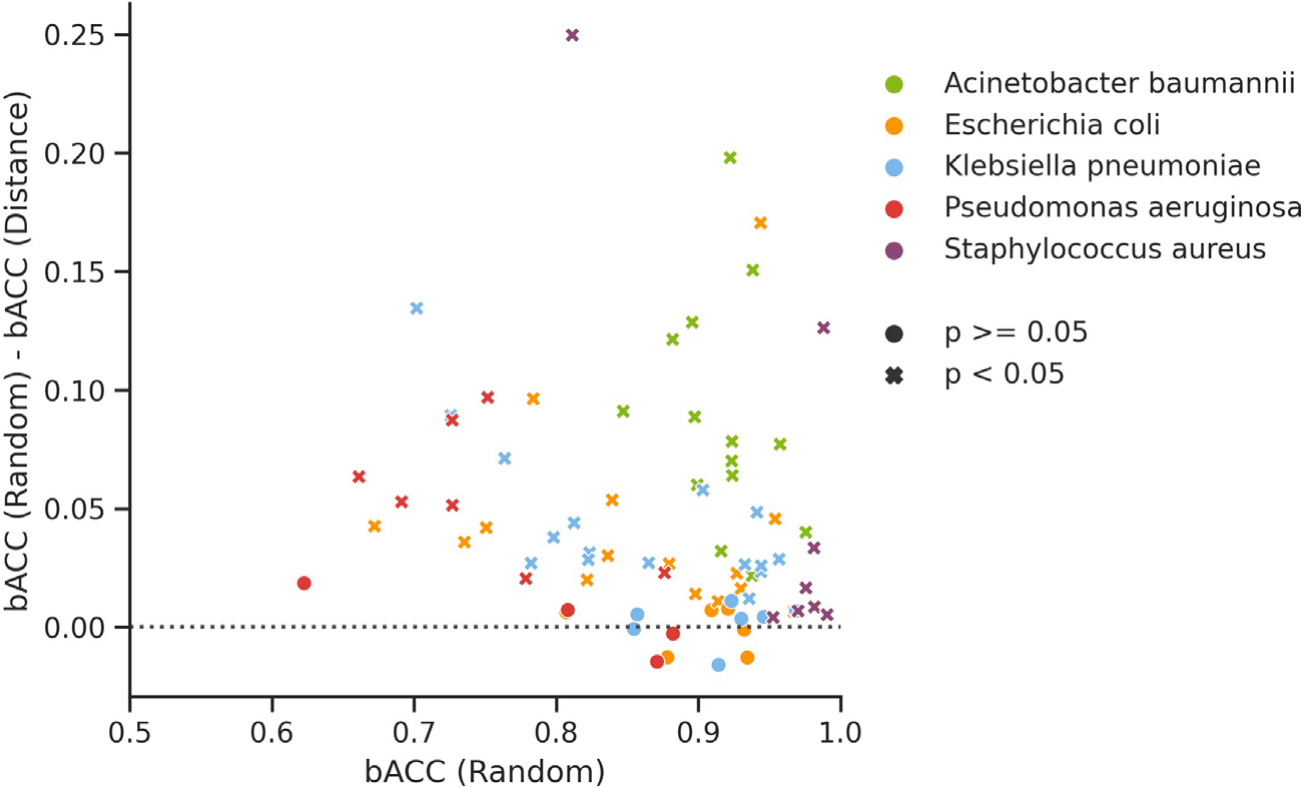
Difference in balanced accuracy (bACC) of XGB models trained and evaluated under random CV and genome distance-aware CV for all considered organism/compound pairs. Significance thresholds are the probability of obtaining bACC estimates as low or lower than the ones from genome distance-aware CV when sampling from a normal distribution fitted to 10 random CV replicates obtained with different random seeds.

Of the 77 investigated organism/compound pairs, 60 exhibited significantly higher balanced accuracy (bACC) estimates for random CV than for genome distance-aware CV (**Figure 1**). The average bACC estimated by random CV was 4.45% greater than that of distance-aware CV, indicating that performance estimates by random CV are likely to overestimate the true performance of WGS-AST models on unseen, independent data sampled from a population that is not comprehensively represented in the training data. The observed effect is congruent with published findings of the generalization properties of WGS-AST models applied to independently sampled data (Hicks et al., 2019). To empirically demonstrate that performance estimates by random CV are prone to be overoptimistic we trained XGB models on the full set of *P. aeruginosa* samples and evaluated them on an independent dataset of 140 samples (Ferreira et al., 2020) (see **Supplementary Figure S1**). On average, bACC of the trained XGB models on this test set was 10.12% lower than estimated by random CV. Distance-aware CV provided more conservative estimates while not completely rescuing the overestimation bias, likely due to novel AMR mechanisms associated with the independent dataset (see *Discussion*).

### Benchmarking of Machine Learning Algorithms for WGS-AST

We selected three machine learning algorithms for prediction of antimicrobial resistance from WGS data represented as nucleotide k-mer profiles: extreme gradient boosting (XGB) (Chen and Guestrin, 2016), elastic net regularized logistic regression (ENLR) (Friedman et al., 2010), and set covering machine (SCM) (Marchand and Shawe-taylor, 2000). All selected algorithms were recently reported to perform well on the WGS-AST task (Aun et al., 2018; Nguyen et al., 2018a; Drouin et al., 2019; Ferreira et al., 2020; Lees et al., 2020).

**Figure 2:**
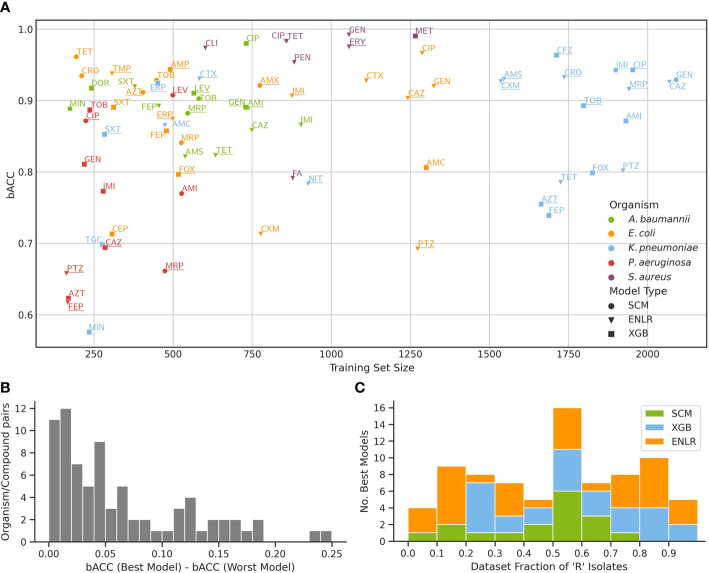
Benchmark of three ML algorithms on the prediction of antimicrobial resistance from WGS data. **(A)** Predictive performance of models for each organism/compound pair as a function of training set size. For each pair, performance of a model with the highest bACC is shown, and underlined if the stacking model outperformed it. The mapping of compound names to compound abbreviations is given in **Supplementary Table S4**. **(B)** Distribution of bACC differences between the models with highest and lowest bACC for all organism/compound pairs. **(C)** Number of top performing models from each algorithm as a function of the fraction of resistant isolates in the training set.

Selected algorithms were benchmarked across a set of five clinically relevant bacterial pathogens and a total of 77 organism/compound combinations (**Figure 2A**). Predictive performance across evaluated algorithms was similar, with a median difference between the strongest and weakest model for an organism/compound combination of 4.22% bACC (**Figure 2B**). ENLR, XGB, and SCM algorithms yielded the model with the highest bACC for 34, 28, and 15 datasets, respectively. Despite their characteristically low complexity and high interpretability, SCM models outperformed the more complex ENLR and XGB models on several datasets, particularly when few resistant isolates were available (**Figure 2C**).

### Model Stacking Improves Predictive Performance and Robustness of Individual ML Models

**Figure 3:**
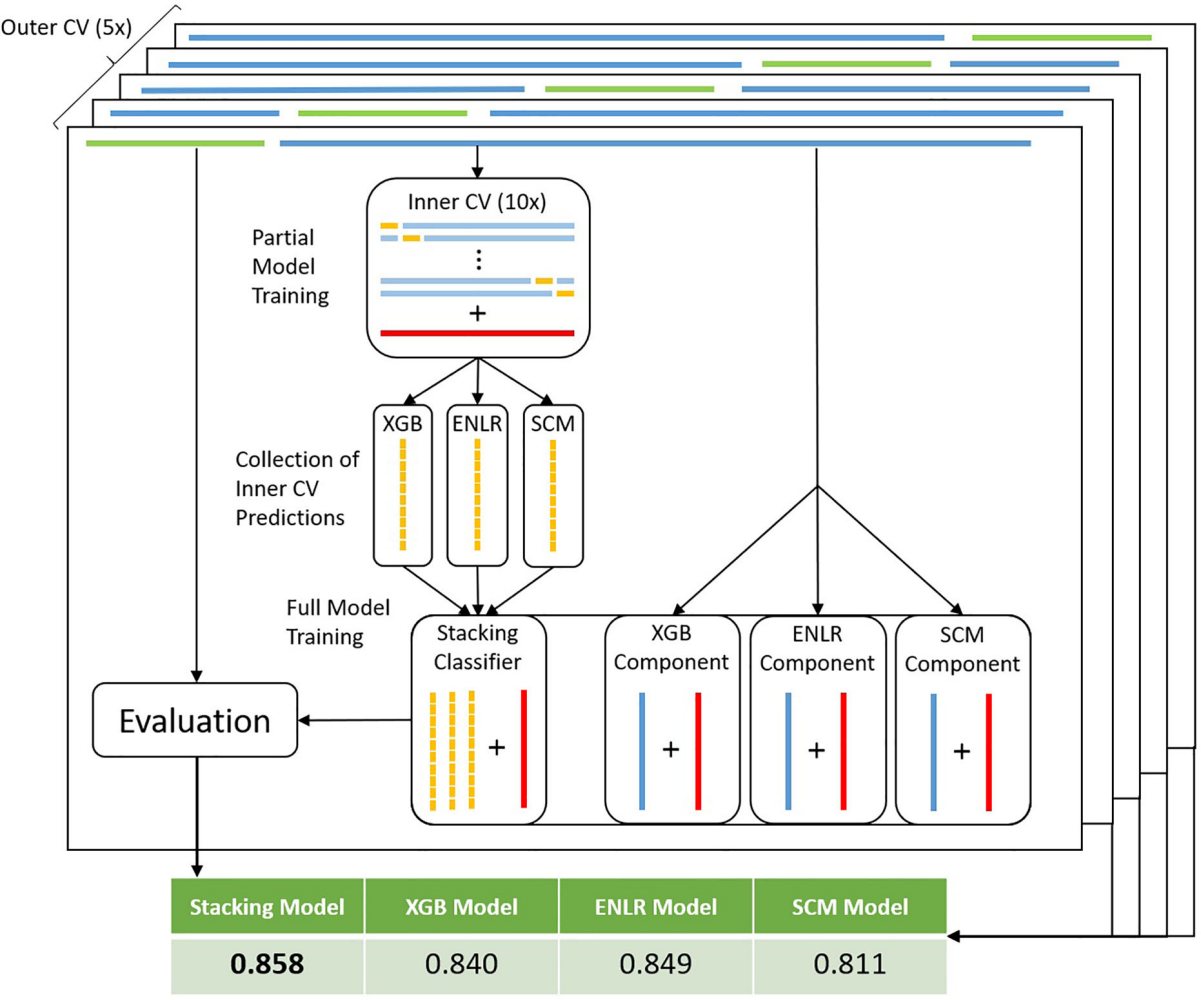
Workflow for model stacking with nested CV. For each training data set in the outer CV loop (dark blue bars on top) complete with true resistance status of samples (red bars), an inner CV loop is run (light blue bars). The full set of predictions (yellow bars) obtained from the test sets of the inner CV are used to train a stacking model to ideally combine predictions from each of the components. At the same time, full component models are trained on the training data set (blue bars within component models). Subsequently, predictions are made by all full component models on the test dataset (green bars on top). Predictions are made by the stacking model using the component model predictions as input features. Finally, performance metrics are obtained by scoring predictions of each model type against the true resistance status of test set samples.

To improve predictive performance, we then employed stacking, a model ensembling technique. The ENLR algorithm was used to train a metamodel which learned to optimally combine predictions of individual component XGB, ENLR and SCM models (**Figure 3** and Methods). We compared the stacked model with a simpler ensembling approach based on the majority vote of all component models. On average, stacked models improved over the sensitivity and specificity of their component models by 1.77% and 3.20%, respectively. The stacking model was found to be the best model by bACC of outer CV in 30 out of 77 organism/compound combinations, outperforming individual component models and the majority vote ensemble. To gauge robustness, we considered a model to have encountered a failure mode if it exhibited a drop in bACC of more than 5.00% compared to the best model for that organism and compound. The stacked models encountered failure modes in 3 out of 77 cases, thus exhibiting superior robustness compared to component models and the simple majority vote ensemble (**Table 1**).

**Table 1:** Summary statistics of model performance (averaged over organisms and compounds) and number of top-1 placements and failure modes (a more than 5% drop in bACC compared to the best performing model) per organism and compound combination.

Algorithm	bACC	Sensitivity	Specificity	# Top-1 Rankings (bACC)	# Failure Modes Encountered
*ENLR*	0.849	0.807	**0.890**	15	9
*XGB*	0.840	0.813	0.866	13	13
*SCM*	0.811	0.805	0.818	8	31
*Majority vote*	0.846	0.818	0.873	17	10
*Stacking*	**0.858**	**0.826**	**0.890**	**30**	**3**

Best metrics in boldface.

### Failure Modes of Component Models and Biological Interpretation

We selected two organism/compound pairs with large differential performance among component models and investigated the biological underpinnings of observed failure modes by annotating k-mers mapping to known AMR biomarkers (Ferreira et al., 2020). For practical reasons, we investigated the models trained in the CV fold exhibiting the largest differential performance and considered only the top 10 most impactful features of each model (see **Supplementary Tables 8** and **9**).

For the combination agent piperacillin and tazobactam (PTZ) in *Klebsiella pneumoniae*, the SCM model exhibited a drop of on average 10% bACC in comparison to XGB and ENLR models. This drop was due to decreased specificity of the SCM model, caused by the model making a comparably larger number of false resistance calls (see **Supplementary Table 6**). Of the four features learned by the model, two mapped to known AMR markers *gyrA* and *catB3*, involved in fluoroquinolone and phenicol resistance, respectively, with no known function in PTZ resistance (Bunny et al., 1995; Drlica and Zhao, 1997). This indicates a strong reliance of the model on features which are spuriously correlated with the phenotype. Conversely, the corresponding XGB model learned multiple k-mers mapping to *blaKPC* beta-lactamase genes, known to confer resistance to piperacillin (Bush and Jacoby, 2010). The stacking model incorporating this SCM model learned to fully disregard the predictions of the SCM model in favor of ENLR and XGB predictions (see **Supplementary Table 7**).

Conversely, for tobramycin (TOB) in *Acinetobacter baumannii*, XGB and ENLR exhibited reduced bACC, mostly due to failure to identify resistant samples in one CV fold. The SCM model performed consistently well. Feature analysis showed that two of the only three features considered by the SCM model could be mapped to N-Acetyltransferase genes *aadB* and *aacC5*, known to confer resistance to aminoglycosides (Shaw et al., 1993; Cox et al., 2015). The XGB and ENLR models learned a high number of features (512 and 6351, respectively), indicating potential overfitting. In the top 10 features of each, only XGB exhibited interpretable features, namely *aacA16*, an aminoglycoside acetyltransferase, and *msrE*, conferring resistance to erythromycin (Sharkey and O’Neill, 2018). The stacking model learned to assign the highest weight to the SCM component, thereby achieving second place performance after the individual SCM itself (see **Supplementary Table 7**).

## Discussion

### Random CV May Overestimate WGS-AST Model Generalizability

We demonstrate on a large collection of public datasets that special care must be taken when applying machine learning techniques to the WGS-AST problem. Two common properties of genomics datasets, namely high dimensionality (Clarke et al., 2008) and sparse and biased sampling of the underlying data distribution, invalidate default design choices such as random dataset partitioning for evaluation of generalizability.

Awareness of the issue of splitting data for WGS-AST ML is developing; a recent study (Aytan-Aktug et al., 2020) used genome clustering based on a similarity threshold, splitting only full clusters into different CV folds together. This approach to data partitioning is also widely used in gene- and protein-based deep learning, where generally only a single training, validation, and test dataset are used (AlQuraishi, 2019; Strodthoff et al., 2019). While grouping by a similarity threshold increases biological meaningfulness and independence of data splits (potentially further reducing performance overestimation), it may cause strongly disbalanced CV fold sizes, especially in a small data regime. The genomic distance-aware method proposed in this work by design generates equally sized folds and aims at maximizing the sample independence across the folds. **Supplementary Figure S3** shows how the proposed method partitions public *P. aeruginosa* samples used in this work.

Similarly, hierarchical clustering has been used for removal of highly clonal genomes from the dataset (Nguyen et al., 2018b), though mainly due to computational considerations. While deduplication is likely to reduce the impact of dependence structures in the training data, the large dimensionality and sparsity of AMR information in a genome represented as k-mer counts makes finding a useful deduplication criterion tricky, especially if the goal is for the model to learn unknown AMR mechanisms.

Of note, data splitting methods controlling for population structure are expected to provide performance estimates differing from random splitting under two conditions: significant population structure must exist in the training dataset, and causal AMR mechanisms must be correlated with population structure. Datasets of closely related samples (not reflecting the true diversity of the underlying population), and datasets containing homogeneously distributed AMR mechanisms, allow only limited insight into possible performance drops due to novel AMR mechanisms associated with distinct populations. Thus, such techniques may still overestimate performance on independently sampled datasets to varying degrees.

Ultimately, a comprehensive assessment of the impact of different clustering and deduplication strategies on model generalizability estimates may be valuable. However, to not only overcome overestimation of performance but to raise predictive accuracy beyond FDA requirements for AST devices (FDA, 2009) and hasten application of WGS-AST models in a diagnostic setting, a greater depth and width of training and test data will be required.

### Benchmarking of Machine Learning Algorithms for WGS-AST

Comparing three different ML algorithms, we find that no single algorithm is clearly superior using the respectively chosen feature space, model parametrization and evaluation criteria. While training set size was positively correlated with performance of all investigated algorithms (see **Supplementary Figure S2**), both species identity and antibiotic compound class clearly influenced classifier performance. Previously established findings regarding the significant challenge in providing accurate AMR predictions for *P. aeruginosa* have been affirmed by this work (Aun et al., 2018). Likewise, we obtain high accuracy predictions for *S. aureus* and most antibiotic compounds in *E. coli*, reflecting earlier results obtained with approaches operating on curated sets of AMR markers instead of nucleotide k-mers (Bradley et al., 2015; Moradigaravand et al., 2018). A notable example of the influence of the compound class on prediction accuracy is the consistently high performance of models for resistance to the fluoroquinolones ciprofloxacin (CIP) and levofloxacin (LEV), which is strongly determined by single nucleotide polymorphisms to the DNA gyrase gene *gyrA* and topoisomerase IV gene *parC* (Jacoby, 2005).

### Model Stacking Improves Predictive Performance and Robustness of Individual ML Algorithms

Several WGS-AST machine learning techniques have been described in the scientific literature. We demonstrate that individual ML algorithms, while performing similarly on average, are susceptible to different failure modes when applied to the WGS-AST problem, such that no single algorithm is clearly preferable for all organism and compound combinations. We illustrate that a stacking ensemble improves predictive performance and robustness, largely beyond that of any of its component models.

It has been suggested that the use of a diverse set of learning algorithms improves predictive accuracy of ensembling models (Kuncheva and Whitaker, 2003). While we systematically benchmarked three algorithms previously reported to perform well on the problem at hand, adding additional ML architectures to the stack is straightforward and may be a promising next step to further improve predictive accuracy and robustness, even in the absence of additional data. Conversely, we note that in settings where model interpretability is of overriding importance, for example in biomarker discovery, individual highly interpretable models such as the SCM may be preferred over complex model ensembles.

### Conclusion

We describe the choice of ML model evaluation strategy and architecture as key aspects affecting model performance and generalizability based on publicly available WGS-AST data sets. To facilitate WGS-AST across organism-compound combinations and translation into clinical practice, applying best practice machine learning techniques and further complementing of publicly available WGS-AST data is important.

## Materials and Methods

### Data Retrieval

Genome assemblies and associated resistance/susceptibility profiles for five clinically relevant pathogens (*A. baumannii, E. coli, K. pneumoniae, P. aeruginosa*, and *S. aureus*) were obtained from public data sources (See **Supplementary Tables 1** and **2**) (Karp et al., ; NCBI NCBI, ; Kos et al., 2015; Wattam et al., 2016; Nguyen et al., 2018a; Mahfouz et al., 2020). Minimum inhibitory concentration (MIC) values, if present, were interpreted (S/I/R) *via* clinical breakpoints according to CLSI 29 standards (Wayne, 2019). Intermediate phenotypes were treated as resistant for model training and evaluation. Isolates with MIC values less than or equal to a dilution step in the intermediate range (meaning that the MIC interpretive category was ambiguous according to CLSI 29 standards) were treated as susceptible. Data was filtered to pass assembly QC metrics (Ferreira et al., 2020). Finally, only organism-compound pairs were included for which at least 50 susceptible and resistant isolates as well as 200 isolates in total could be retrieved (see **Supplementary Tables 1–3**). Using these cut-offs, a total number of 8704 genome assemblies were retrieved.

Genome assemblies used for evaluation of CV estimates on an independent dataset (Ferreira et al., 2020) were obtained from NCBI (PRJNA553678). AST data were obtained from the authors.

### Data Partitioning for Training and Evaluation

Models were trained and evaluated in a nested 10x/5x cross-validation scheme, whereby the inner 10x cross-validation was used to obtain the training features for the stacking model (**Figure 2**).

Genome-distance-based cross-validation folds were created for each species individually such that genome distance was maximized between the test sets of folds (see **Supplementary Methods Section 1**). In short, for all assemblies of each organism, a distance matrix was computed with Mash v2.2 (Ondov et al., 2016). From the distance matrix, two seed samples with the largest genomic distance among them were identified. Subsequently, for each remaining sample, the minimal distance to either of the seeds was computed. Additional seed samples up to the number of desired CV folds were added by selecting samples with the highest minimal distance to existing seeds. Finally, all remaining samples were assigned to seed samples iteratively by assigning to each seed the sample with the lowest genomic distance. The generated five sample groups of even size were used as input to CV. Randomly split CV folds for comparison were created using scikit-learn (Pedregosa et al., 2011).

### Feature Creation and Feature Selection

For XGB and ENLR models, feature extraction and selection were performed according to the following procedure. For all training assemblies of each organism, a count matrix of overlapping k-mers of length 15 was built using KMC 3.1.0 (Kokot et al., 2017). Zero-variance k-mers were removed. Out of all k-mers having identical count profiles across training isolates, only a single representative k-mer was retained. Subsequently, for each organism and relevant antimicrobial compound, a subset of the organism’s full count matrix for which S/R class information of the given compound was available was extracted. The k-mer feature space was then condensed by univariate feature selection before application of machine learning. K-mers were tested for independence from the S/R category using the χ^2^ test as implemented in scikit-learn and filtered by a p-value of p < 0.05. Of the k-mers passing this filtering step, at most 1.5 million k-mers with the highest log-odds ratio were retained. For SCM models, k-mer features of length 31 were created from assemblies with Kover2 according to the supplied manual. To exclude the possibility of biases introduced by common feature selection on the full dataset, features for prediction on the test sets of the outer cross-validation were created only at prediction time.

### Model Training

We trained extreme gradient boosting (XGB), elastic net regularized logistic regression (ENLR) and set covering machine (SCM) models for prediction of antimicrobial susceptibility from WGS data for a set of five clinically relevant pathogens. A fixed set of hyperparameters was used across all organisms and compound pairs, except for the number of trees in the model which was tuned *via* internal CV. We explored the choice of CV method for hyperparameter optimization and found that the performance estimated by the outer CV method is relatively insensitive to the choice of the inner CV method (see **Supplementary Figure S4**) and thus used a distance-based splitting criterion for internal CV of both XGB and ENLR methods. ENLR models were trained using the glmnet_python package, version 0.2.0 (Friedman et al., 2010), and the hyperparameters lambda and alpha were tuned *via* an internal CV. Set covering machine models were trained with the Kover2 package, version 2.0.3 (Drouin et al., 2019) according to the supplied manual and using risk-bound hyperparameter selection (see **Supplementary Methods Sections 4 **and** 5**).

Individual models were combined into a stacked model (Wolpert, 1992), with ENLR serving as the learning algorithm. Classically, stacking is achieved using a disjunct mixing set, whereby the predictions of component models on the mixing set serve as the input features on which the stacking classifier is trained. Due to the limited amount of available data, this was achieved here by training partial component models in an inner 10x (distance-based) CV loop (**Figure 3**). Predictions of component models on all test sets were then concatenated into the training features of the stacking model. Predictions with the stacked model were made on the prediction output of the individual, full component models (XGB, ENLR, and SCM) (see **Supplementary Methods Section 2**).

### Model Evaluation

Component ML models as well as the stacking model were evaluated in the outer CV loop by predicting the MIC interpretive category (susceptible or resistant) on samples in the test set. Confusion matrices were summed up from outer CV folds. Performance of trained models was evaluated on the balanced accuracy (bACC) metric (Brodersen et al., 2010), as this metric allows evaluation of a model on imbalanced datasets. The bACC is furthermore related to the arithmetic mean of very major error (VME) and major error (ME), two performance criteria commonly applied to AST testing methods. Models created by the individual algorithms (XGB, ENLR, SCM), the majority vote ensemble model and the stacking model were ranked by counting the number of other models achieving higher bACC on each organism/compound pair.

## Data Availability Statement

The original contributions presented in the study are included in the article/**Supplementary Material**. Further inquiries can be directed to the corresponding author.

## Author Contributions

LL, PM, SB, TR, and AP devised the study design. LL and PM wrote the code, performed experiments, and analyzed the resulting data. LL wrote the first draft of the manuscript. LL, PM, and SB wrote sections of the manuscript. All authors contributed to the article and approved the submitted version.

## Funding

This work was supported by the Austrian Research Promotion Agency (FFG) (grants 866389, 874595, and 879570).

## Conflict of Interest

LL, PM, SB, and AP are employed by Ares Genetics GmbH.

The remaining author declares that the research was conducted in the absence of any commercial or financial relationships that could be construed as a potential conflict of interest.
