# Supplementary material for: Learning From Limited Data: Towards Best Practice Techniques for Antimicrobial Resistance Prediction From Whole Genome Sequencing Data
Source: Front Cell Infect Microbiol. 2021 Feb 15;11:610348. doi: 10.3389/fcimb.2021.610348 (PMC7917081; doi:10.3389/fcimb.2021.610348)
Supplement: Supplementary file 1 [file DataSheet_1.docx]

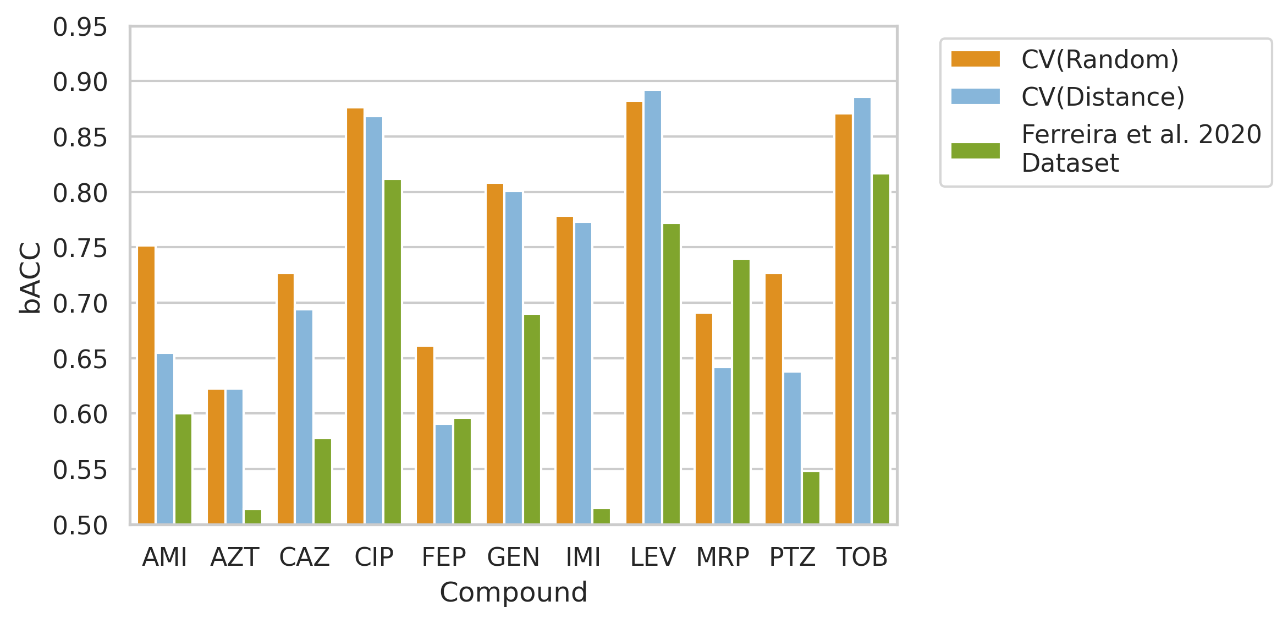


Figure S1. Performance of *P. aeruginosa* XGB models estimated during random or distance-based CV, as well as performance found when applied to an independent dataset of 140 samples from Ferreira et al. 2020.


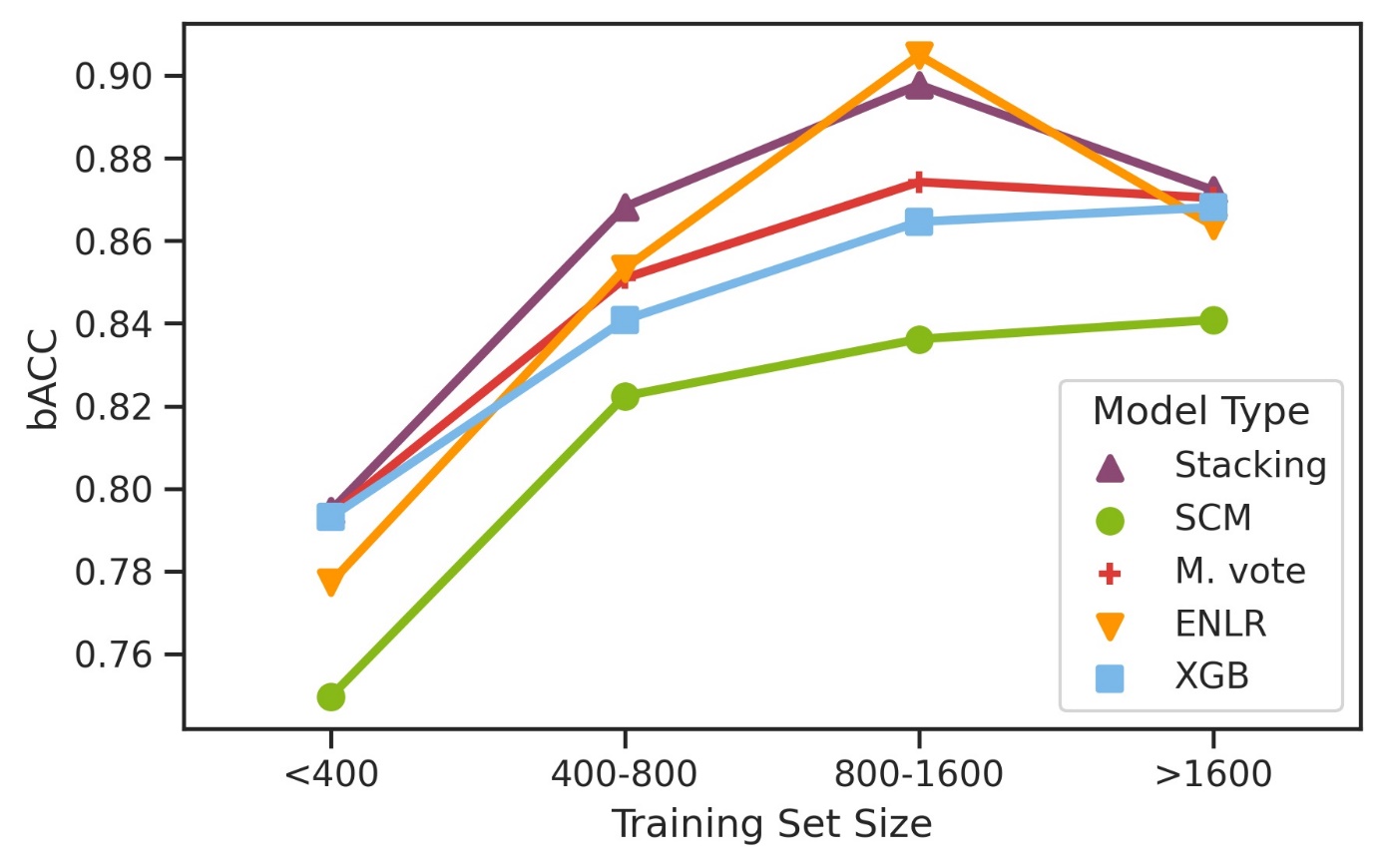


Figure S2. Performance of individual and ensemble WGS-AST models (averaged over organisms, compounds and outer CV folds) as a function of training set size.


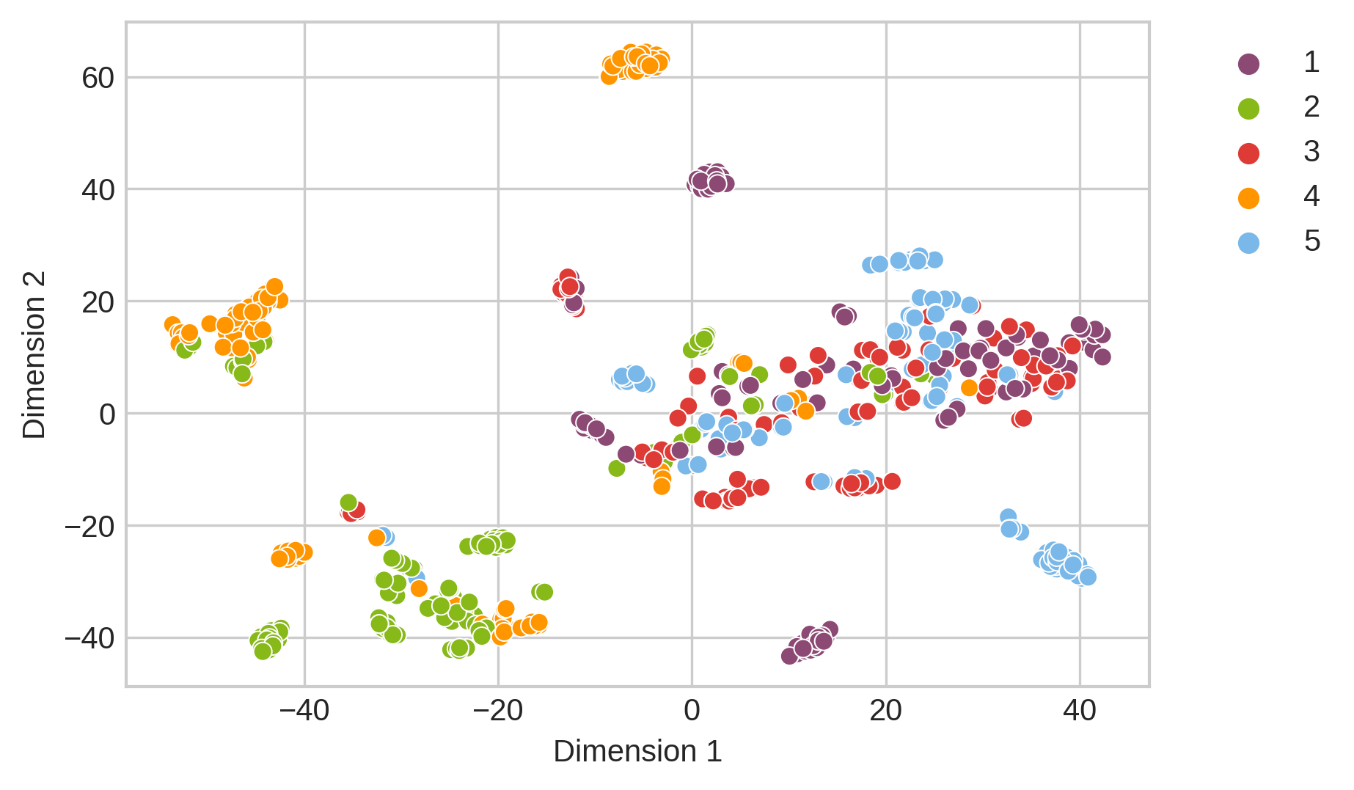


Figure S3. t-SNE embedding of genome distances for *P. aeruginosa* samples used in this work, coloured by the group assignment of distance split used for determining the test set in distance-based CV.


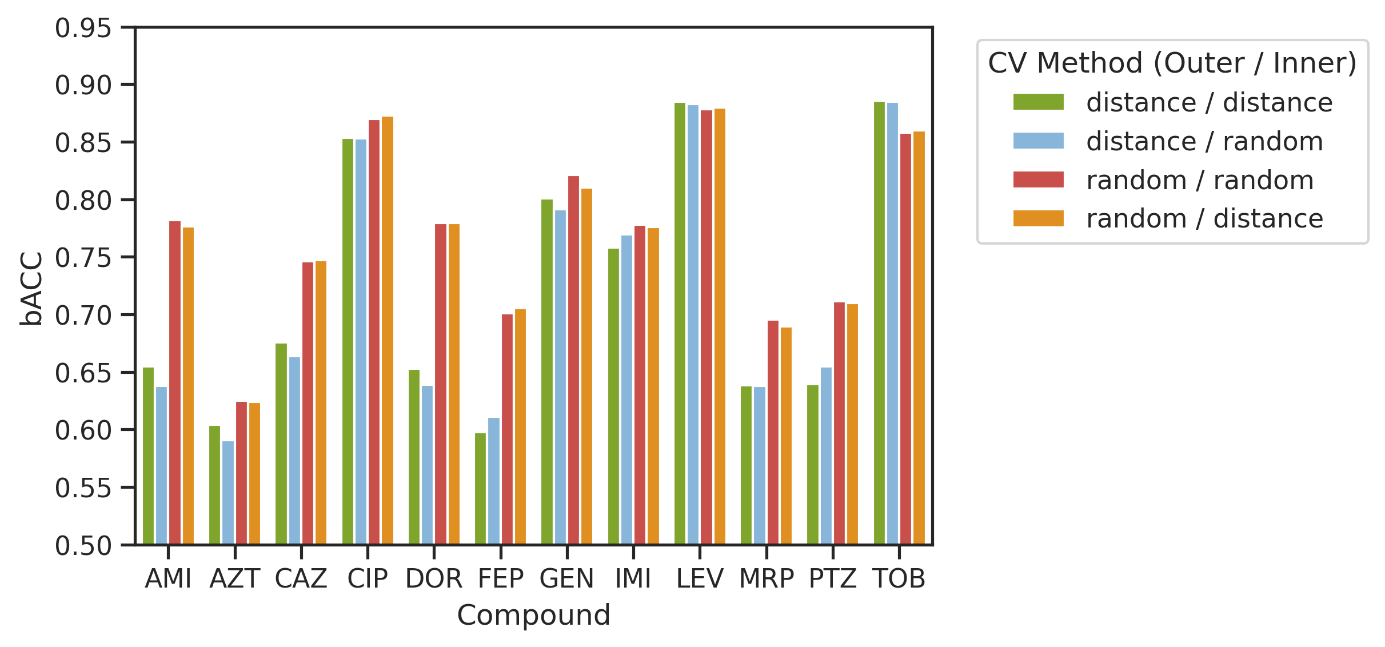


Figure S4. Effect of the use of distance-based or random split CV in inner CV loop (used to search the hyperparameter maximum tree depth) and outer CV loop (used for evaluation of trained models) of *P. aeruginosa* XGB models.
