# Supplementary material for: Learning From Limited Data: Towards Best Practice Techniques for Antimicrobial Resistance Prediction From Whole Genome Sequencing Data
Source: Front Cell Infect Microbiol. 2021 Feb 15;11:610348. doi: 10.3389/fcimb.2021.610348 (PMC7917081; doi:10.3389/fcimb.2021.610348)
Supplement: Supplementary file 2 [file DataSheet_2.docx]

# Genome Distance Splitting Algorithm

**Algorithm: Greedy distance informed dataset partitioning algorithm**

**Inputs:** ***S*** a set of samples, ***D*** a distance matrix of all samples in ***S***, ***k*** a number of subsets to return

**Output:** a partition of ***S*** into ***k*** equally sized subsets (|***S***| *mod* ***k*** subsets are larger by 1 sample)

#1. Find initial seeds of *K* subsets with maximal distance

*a*, *b* ≔ *argmax*{*D_i,j_* : ∀*i*,*j* ϵ *S* }

*F(a)* ≔ *0*; *F(b)* ≔ *1*

*Seeds* ≔ {*a*, *b*}; *S ≔ S – Seeds*

**while** |*Seeds*| *< k* **do**

∀*x* ϵ *S* **set** A(x) ≔ *min*{*D_x,y_* : ∀*y* ϵ *Seeds*}

*c* *≔* *argmax{*A(x) : ∀*x* ϵ *S* }

*F(c) ≔* |*Seeds*|

*Seeds ≔ Seeds* ∪ *{c}; S ≔ S – {c}*

**end while**

#2. Iteratively grow each subset

*i ≔ 0* # *i* is the subset to grow next

**while** |*S>0*| **do**

∀*x* ϵ *S* **set** A(x) ≔ min{D_x,y_ : ∀y s.t. *F(y)=i*}

*c ≔ argmin*{*A(x)* : ∀*x* ϵ *S* } # find the closest sample to the samples in the subset *i*

*F(c) ≔ i*; *S ≔ S – {c}*; #assign *c* to the subset *i*

*i ≔ (i+1) mod k* # proceed with the next subset

**end while**

**return** *F*

# Stacking

The individual models *M_i_* were combined into a stacked model (Wolpert, 1992) via the following sigmoid function

$M(x)=f(w_{0}+ \sum_{i=1..N} w_{i}g(M_{i}(x)))$ (1)

where *f* is the logistic function $f(x)=\frac{1}{1+e^{-x}}$ and

$g\left( M_{i}\left( x \right) \right)=\left\{ \begin{matrix} f^{-1}\left( M_{i}\left( x \right) \right) \mathrm{if} M_{i} is a probability returning model \\ M_{i}\left( x \right) \mathrm{otherwise} \end{matrix} \right.$. (2)

To determine optimal weights $w_{i}$ of the stack model (1) a labeled mixing set is required. To avoid overfitting, the samples of the mixing set must not be used in the training of individual models *M_i_*. Classically, this is accomplished by splitting the available labeled data into a training set and a mixing set. However, having a limited set of labeled data available it is desirable to use all available data for training of individual models as well as for learning the mixing weights. This can be accomplished by approximating $M_{i}\left( x \right)$ in Eq. (1) with the prediction of a partial model $M_{i}'(x)$ such that the sample *x* was not used in the training of $M_{i}'$. Such partial models $M_{i}'(x)$ can be obtained by K-fold cross validation; K=10 was used in the presented work. First, full models of each type were trained on all training samples. Then the training set was split via distance informed partitioning algorithm into 10 folds. Next, 10 partial models were trained, each on a different 90% subset of the training data. Predictions of each partial model on the remaining 10% of the data were recorded and concatenated to form a vector of out-of-fold (OOF) predictions$M_{i}'(x)$. The assumption is that a partial model trained on 90% of the data is a close approximation of the full model. The concatenated OOF predictions $M_{i}'(x)$ were then used to learn optimal weights of the stack model in Eq. (1). Each model was thus trained 11 times (once on the full training set and 10 times to generate OOF predictions). As a result, all training samples were utilized for training of individual models as well as for learning the stacking weights $w_{i}$. The weights of the ensemble in Eq. (1) were trained on the generated OOF predictions using the elastic network algorithm (Friedman et al., 2010) with hyperparameters α and λ optimized by internal cross validation of the glmnet_python package.

# Significance Calculation of Random vs distance-aware CV

# The variance of random CV was estimated by executing the method 10 times for each organism/compound combination, each time using a different random seed. To obtain statistical significance measures of performance differentials between random and genome distance-aware CV, it was assumed that random CV estimates are normally distributed with the mean and SD as estimated by the 10 replicated experiments. The p-value was then given as the probability of obtaining a bACC as low or lower than the one obtained from genome distance-aware CV, when sampling from the thus obtained normal distribution.

# Genome sizes used for risk bound hyperparameter selection in Kover2

| Species | Genome Size (Mbp) |
| --- | --- |
| *Acinetobacter baumannii* | 4 |
| *Escherichia coli* | 5.6 |
| *Klebsiella pneumoniae* | 5.7 |
| *Pseudomonas aeruginosa* | 6.3 |
| *Staphylococcus aureus* | 2.9 |

# Hyperparameters used for XGB models

| eval_metric | logloss |
| --- | --- |
| objective | binary:logistic |
| colsample_bytree | 0.8 |
| subsample | 0.8 |
| learning_rate | 0.05 |
| max_depth | 4 |
| min_child_weight | 1 |
| scale_pos_weight | # susceptible training samples / # resistant training samples (capped at 0.1 and 10) |

# Binary Classification Metrics

| Name | Formula |
| --- | --- |
| True Positive | Model correctly predicts resistance |
| False Negative | Model wrongly predicts susceptibility |
| False Positive | Model wrongly predicts resistance |
| True Negative | Model correctly predicts susceptibility |
| TN, FN, FP, TP | (number of predicted instances which were) True Negative, False Negative, False Positive, True Positive |
| Accuracy | (TN + TP) / (TN + FN + FP + TP) |
| Sensitivity | TP / (TP + FN) |
| Specificity | TN / (TN + FP) |
| Balanced Accuracy (bACC) | - 1. * (Sensitivity + Specificity) |
